# Relationship of the adherence to the Mediterranean diet with health-related quality of life and treatment satisfaction in patients with type 2 diabetes mellitus: a post-hoc analysis of a cross-sectional study

**DOI:** 10.1186/s12955-016-0473-z

**Published:** 2016-05-04

**Authors:** Nuria Alcubierre, Montserrat Martinez-Alonso, Joan Valls, Esther Rubinat, Alicia Traveset, Marta Hernández, Maria Dolores Martínez-González, Minerva Granado-Casas, Carmen Jurjo, Jesus Vioque, Eva Maria Navarrete-Muñoz, Didac Mauricio

**Affiliations:** Institut de Recerca Biomèdica de Lleida, University of Lleida, Lleida, 25198 Spain; Biostatistics Unit, Institut de Recerca Biomèdica de Lleida, University of Lleida, Lleida, 25198 Spain; Unitat de Suport a la Recerca de Barcelona, Institut Universitari d’Investigació en Atenció. Primària Jordi Gol (IDIAP Jordi Gol), CIBER of Diabetes and Associated Metabolic Diseases (CIBERDEM), Barcelona, 08007 Spain; Department of Ophthalmology, University Hospital Arnau de Vilanova, Lleida, 25198 Spain; Department of Endocrinology and Nutrition, University Hospital Arnau de Vilanova, Lleida, 25198 Spain; Consortium for Biomedical Research in Epidemilogy and Public Health (CIBER en Epidemiología y Salud Pública CIBERESP), Madrid, 28029 Spain; Public Health Department, Miguel Hernandez University, Alicante, Spain; Department of Endocrinology and Nutrition, University Hospital and Health Sciences Research Institute Germans Trias Pujol, CIBER of Diabetes and Associated Metabolic Diseases (CIBERDEM), Badalona, 08916 Spain

**Keywords:** Type 2 diabetes mellitus, Relative Mediterranean Diet score, Quality of life, Treatment satisfaction, Mediterranean diet

## Abstract

**Objective:**

The main aim of this study was to assess the association between adherence to the traditional Mediterranean diet (MedDiet) and health-related quality of life (HRQoL) and treatment satisfaction in patients with type 2 diabetes mellitus (T2DM).

**Methods:**

This cross-sectional study included 294 patients with T2DM (146 with diabetic retinopathy and 148 without retinopathy). HRQoL and treatment satisfaction were assessed with the Audit Diabetes-Dependent Quality of Life and Diabetes Treatment Satisfaction Questionnaires, respectively. Adherence to the MedDiet was evaluated with the relative Mediterranean Diet Score (rMED). The rMED was added to multivariate linear regression models to assess its relative contribution as a quantitative as well as a qualitative variable after recoding to maximize each of the model’s coefficients of determination to explain quality of life as well as treatment satisfaction dimensions.

**Results:**

The adherence to the Mediterranean diet showed no significant association with the overall quality of life score. However, rMED was associated with some HRQoL dimensions: travels, self-confidence and freedom to eat and drink (*p* = 0.020, *p* = 0.015, *p* = 0.037 and *p* = 0.015, respectively). Concerning treatment satisfaction, rMED was positively associated with its overall score (*p* = 0.046), and especially with the understanding of diabetes (*p* = 0.0004) and treatment recommendation (*p* = 0.036), as well as with the perceived frequency of hyperglycaemias (*p* = 0.039).

**Conclusion:**

Adherence to the Mediterranean diet was associated with greater treatment satisfaction in patients with T2DM. Although we found no association with overall HRQoL, adherence to this dietary pattern was associated with some quality of life dimensions.

## Background

Despite the fact that there is increasing scientific evidence on the role of the traditional Mediterranean Diet (MedDiet) as an indicator of physical and mental health and of healthy ageing in several population subgroups (adolescents, university students, general population, elderly people, and subjects with high blood pressure), there are very few data available on the relationship of the food intake pattern with quality of life and treatment satisfaction in patients with type 2 diabetes mellitus (T2DM) [1–6]. Kahleova et al. assessed the impact of two low calorie diet interventions (vegetarian diet vs. conventional diabetes diet), to which a programme of aerobic exercise was added, on health-related quality of life (HRQoL), eating behaviour and mood of obese patients with T2DM [7]. The results showed the positive effect of a low calorie vegetarian food regime on the quality of life and mood of these patients [7]. A recent sub-analysis of the PREDIMED trial demonstrated the role attributable to the Mediterranean diet regime, enriched with nuts, in the prevention of unipolar depression in patients with T2DM [8].

In T2DM, maintaining quality of life and treatment satisfaction are important treatment goals for the patient and are increasingly important in health decision-making [9, 10]. Both outcome variables demonstrated their association with lower morbidity and mortality, greater treatment adherence and positive changes in lifestyle-related aspects [10, 11].

Medical nutrition therapy, as an integral part of diabetes education, is a cornerstone in the comprehensive approach to diabetes [12]. The implementation of interventions which facilitate a change of lifestyle is fundamental to achieve the therapeutic goals and delay its advanced complications [13]. The MedDiet is a reference as a healthy eating pattern and is also a suitable dietary approach for the management of diabetes and its associated cardiovascular risk factors [14].

In view of all the above, we hypothesized that greater adherence to the MedDiet by patients with T2DM is associated with greater quality of life and treatment satisfaction. The aim of this study was to explore the association between adherence to the MedDiet and quality of life and treatment satisfaction in patients with T2DM. Additionally, we also analyzed the relationship between each component of the Audit of Diabetes Dependent Quality of Life (ADDQoL-19) and Diabetes Treatment Satisfaction Questionnaire (DTSQ) with adherence to the traditional Mediterranean diet (rMED).

## Methods

### Study design and population

We used the data obtained in a cross-sectional study designed with the aim of assessing the differences in terms of HRQoL and treatment satisfaction in patients with T2DM with retinopathy. Five out of the 299 participants in the initial study declined to participate in the nutritional assessment. Therefore, this post-hoc analysis included 294 participants from the previous study. The results of that study have already been communicated in a previous publication [15]. A detailed description of the study design and methodology, and of the patient characteristics has therefore already been provided. In the previously published study on HRQoL and treatment satisfaction, we were able to demonstrate that the presence and severity of retinopathy, duration of diabetes and treatment with insulin were associated with worse scores in HRQoL. Treatment satisfaction was affected by diabetes duration, treatment with insulin and the severity of macular oedema [15]. None of the patients included had prior cardiovascular complications or other advanced complications of diabetes. All participants signed an informed consent form. The study was approved by the local Ethics Committee.

### Health-related quality of life

To assess HRQoL, we used the Spanish version of the specific quality of life questionnaire for diabetic patients, the Audit of Diabetes-Dependent Quality of Life (ADDQoL-19), specifically designed to measure the individual perspective of the impact of diabetes and its treatment on quality of life [16]. The first two items are of a general in nature and are scored separately. The first one assesses current quality of life, with scores going from −3 (extremely bad) to +3 (excellent), and the second one analyzes the overall impact of diabetes on quality of life, with scores going from −3 (maximum negative impact of diabetes) to +1 (maximum positive impact of diabetes). The individual items include questions about 19 specific areas of life. This questionnaire allows a weighted final score of the effects of diabetes and its treatment on patient quality of life, which ranges from −9 (maximum negative impact of diabetes) to +3 (maximum positive impact of diabetes) [17].

### Treatment satisfaction

To measure the satisfaction with treatment we used the specific Diabetes Treatment Satisfaction Questionnaire (DTSQ), designed with the aim of assessing the degree of treatment satisfaction in the adult patients with type 1 and type 2 diabetes [18]. Its use has been recommended by the World Health Organization and the International Diabetes Federation, and it has been validated in the Spanish population [19, 20]. The DTSQ-s questionnaire consists of 8 items which allow 7 possible answers, which range from 0 (very dissatisfied) to 6 (extremely satisfied) points each. On adding 6 of the 8 items we obtain an overall satisfaction score which ranges from 0 points (least satisfaction possible expressed by means of the questionnaire) to 36 points (greatest satisfaction possible expressed by means of the questionnaire). The two remaining items, which refer to the frequency of episodes of hyperglycaemias and hypoglycaemias perceived by the patient, can range from 0 (never) to 6 (most of the time), and are analyzed in an individual and descriptive manner. Higher scores are indicative of higher levels of current treatment satisfaction [18]. All the questionnaires were administered by trained personnel.

### Dietary data

Daily food and nutrient intake was assessed using a semi-quantitative food frequency questionnaire of 101 items, validated for the Spanish population (available at: http://bibliodieta.umh.es/cfa-101-inma) [21]. Its use allowed us to obtain the daily nutrient intake and the food quality index, the relative Mediterranean Diet score (rMED) [22]. The rMED food quality index allowed us to assess the level of adherence to the MedDiet on a linear scale of 18 points. All the components, except for alcohol, were assessed in grams per 1,000 kcal, with the aim of expressing intake in terms of caloric density. These components were weighted with values from 0 to 2, taking as a reference the population tertiles previously published [21]. Adherence was rated as low (0–6), intermediate (7–10) and high (11–18) [22].

### Physical activity

To determine the level of physical activity, we used the active leisure time concept developed by Bernstein et al., which defines a sedentary person as one who invests less than 10 % of the daily energy expenditure in any kind of physical activity which requires at least 4 METs (degree of physical activity equal to or greater than the expenditure of walking at a fast pace for 30 min) [23].

### Statistical analysis

A bivariate analysis between rMED and each of the dimensions of the quality of life and treatment satisfaction categories was performed by using Pearson and Spearman correlation coefficients and, after recoding rMED into categories to assess non-linear associations, by using the Mann-Whitney *U* test (in case of only 2) or Kruskal-Wallis test (for differences among the three levels of rMED). For those dimensions with a significant bivariate association with rMED, multivariate linear regression models were adjusted by adding rMED to the previously published model for global quality of life and treatment satisfaction scores [15]. Non-significant variables in multivariate models were dropped from the final model unless having shown a confounding effect or significant interaction with another variable. The language and environment for statistical computing known as R was used as statistical software. A significance level of 0.05 was established.

## Results

Table 1 shows the clinical and socio-demographic characteristics and the relative Mediterranean Diet score of the study population.

**Table 1:** Clinical and demographic characteristics of the study groups

Characteristics	No DR (*n* = 148)	DR (*n* = 146)	*p*-value
Age (years), mean (SD)	57.9 (10.3)	60.5 (8.8)	0.021
Sex, men, n (%)	77 (52 %)	73 (50 %)	0.816
Ethnicity, non caucasian, n (%)	5 (3.4 %)	6 (4.1 %)	0.769
HbA1c (%), mean (SD)	7.3 (1.2)	8.3 (1.4)	<0.001
Diabetes duration (years), mean (SD)	7.1 (5.5)	14.1 (9.9)	<0.001
Waist (cms), mean (SD)	104.2 (11.9)	107.8 (11.9)	0.010
Systolic blood pressure (mmHg), mean (SD)	134.3 (15.5)	145 (19.9)	<0.001
Diastolic blood pressure (mmHg),mean (SD)	76.5 (10.5)	77.1 (11.1)	0.628
Total cholesterol (mg/dL), mean (SD)	185.7 (36.6)	184.8 (36.1)	0.822
HDL-cholesterol (mg/dL), mean (SD)	48.5 (10.7)	52.1 (15)	0.021
LDL-cholesterol (mg/dL), mean (SD)	111 (30.7)	106.9 (30.1)	0.182
Triglycerides (mg/dL), mean (SD)	138.6 (81.8)	140.5 (120)	0.879
Diabetes treatment			<0.001
Oral agents	95 (64.2 %)	64 (43.8 %)	
Insulin ± oral agents	17 (11.5 %)	79 (54.1 %)	
Diet	36 (24.3 %)	3 (2.1 %)	
Educational level, n (%)			0.629
≤ Primary	90 (60.8 %)	113 (77.4 %)	
Secondary	40 (27.0 %)	30 (20.5 %)	
Graduate or higher	18 (12.2 %)	3 (2.1 %)	
Physical activity, n (%)			0.045
More than 25 min/day	98 (66.2 %)	81 (55.9 %)	
Less than 25 min/day	50 (33.8 %)	64 (44.1 %)	
rMED	8.3 (2.9)	7.7 (2.8)	0.220

*DR* diabetic retinopathy, *HbA1c* glycated haemoglobin, *HDL* high density lipoprotein, *LDL* low density lipoprotein, *rMED* relative Mediterranean Diet score, *SD* standard deviation. Differences between groups were analyzed by Chi-squared or Fisher's exact text (qualitative variables) or Student's test (quantitative variables)

The bivariate analysis revealed that rMED was significantly associated with the following HRQoL dimensions: travels (*p* = 0.025 for differences among the three levels of rMED), self-confidence (*p* = 0.006 for differences among the two groups defined by rMED median), freedom to eat (*p* = 0.030 for differences among the two groups defined by rMED median), and freedom to drink (*p* = 0.005 for differences among the three levels of rMED). Table 2 shows the results of a multivariate regression model to explain the impact of adherence to the MedDiet on the above mentioned items (“travels”, “self-confidence”, “freedom to eat” and “freedom to drink”). An intermediate-high rMED level was negatively associated with the score in the item that assesses ease of travel (*p* = 0.020).

**Table 2:** Multivariate linear regression for dimensions from the Audit of Diabetes Dependent Quality of Life

Coefficients	rMED	Estimate (SE)	*p*-value
Travel^a^	>7	−0.450 (0.192)	0.020
Self-confidence^b^	>8	0.428 (0.175)	0.015
Freedom to eat^c^	>8	0.839 (0.402)	0.037
Freedom to drink^d^	>8	1.150 (0.471)	0.015

^a^Adjusted for insulin treatment, retinopathy, diabetes duration and the interaction diabetes duration*DR* insulin treatment. ^b^Adjusted for insulin treatment, retinopathy, diabetes duration, age (>65 years), waist and the interaction diabetes duration*DR* insulin treatment. ^c^Adjusted for insulin, diabetes duration and ethnicity. ^d^Adjusted for insulin treatment and ethnicity

*DR* diabetic retinopathy, *rMED* relative Mediterranean Diet score, *SE* standard error

On the contrary, a value in the rMED of 8 or above was positively associated with the score in “self-confidence” (*p* = 0.015), and the score in “freedom to eat” (*p* = 0.037); a high rMED level was positively associated with “freedom to drink” (*p* = 0.015). The analysis did not show any significant contribution of the rMED to explain the variability in other quality-of-life dimensions or in the final HRQoL score.

In the bivariate analysis, the rMED was associated with the final treatment satisfaction score (*p* = 0.027 for differences among the three levels of rMED) and with the following treatment satisfaction dimensions: “hyperglycaemias frequency perception” (*p* = 0.006 for trend among the three levels of rMED), “understanding of diabetes” (linearly correlated with rMED with r = 0.23, *p* = 0.0001), and “treatment recommendation” (*p* = 0.047 for differences among the three levels of rMED). Table 3 shows the results of the multivariate regression model to explain general treatment satisfaction after including adherence to the MedDiet. The rMED score showed a highly significant positive linear correlation with “understanding of diabetes” (*p* = 0.0004).

**Table 3:** Multivariate linear regression for dimensions from Diabetes Treatment Satisfaction Questionnaires

Coefficients	rMED	Estimate (SE)	*p*-value
Final score^a^	>11	2.107 (1.052)	0.046
Hyperglycemias frequency perception^b^	≥9	0.372 (0.179)	0.039
Understanding of diabetes^c^	≥9	0.163 (0.45)	<0.001
Treatment recommendation^d^	≥11	0.823 (0.390)	0.036

^a^Adjusted for insulin treatment, retinopathy, diabetes duration, physical activity (>20 min), smoking. and the interaction between diabetes duration*DR. ^b,c^Adjusted for physical activity (>20 min). ^d^Adjusted for retinopathy, diabetes duration and the interaction between diabetes duration*DR

*DR* diabetic retinopathy, *rMED* relative Mediterranean Diet score, *SE* standard error

The “hyperglycemias frequency perception” was positively associated with rMED values of 9 or above (*p* = 0.039). The specific dimension “treatment recommendation” (i.e., would recommend this form of treatment to someone with a diabetes similar to yours) was associated with high levels of rMED (*p* = 0.036). High levels of rMED significantly contributed to explain higher values in overall treatment satisfaction (*p* = 0.046). The analysis did not demonstrate any other significant contribution between any of the other dimensions of DTSQ and the rMED.

## Discussion

The current study indicates that a high adherence to the MedDiet is associated with higher levels of overall treatment satisfaction in patients with T2DM, and also with some specific dimensions of this outcome variable (perception of hyperglycaemias, understanding and recommend to others). Moreover, these results show that an intermediate-high level of adherence to the MedDiet is associated with some dimensions of an important outcome variable for diabetic patients, i.e., HRQoL.

Despite the fact that recent observational studies demonstrate the association that some components of the MedDiet (antioxidants, fatty acid content and fibre intake) have with the health status of the general population, there is an important knowledge gap in this area [2]. Only a recent sub-analysis of the PREDIMED trial demonstrated the role attributable to the Mediterranean diet regime (enriched with nuts) in the prevention of unipolar depression in patients with T2DM. A 40 % reduction was observed in the risk of developing depression in patients receiving Mediterranean diet in relation to the control group [8]. In diabetic patients participating in that study, the results did not achieve significance when this approach was used with the MedDiet enriched with olive oil. Although the current results suggest the potential impact of adherence to the Mediterranean diet pattern on quality of life of the diabetic patient, we could not find epidemiological evidence on this association. We cannot consider that the results of mental health status are comparable with those of quality of life, although we can interpret that there is probably some interaction between them. Also, despite the fact that the MedDiet, as an integral part of medical nutrition therapy of the patient with T2DM, contributes to the control of metabolic parameters, to the best of our knowledge this is the first study showing a relationship of the adherence to the MedDiet with an outcome variable as important in the field of diabetes as overall treatment satisfaction.

This study has some limitations. First, its design as a cross-sectional observational study did not allow us to establish causal relations, but rather only relations of association. Second, the partial representativeness of the sample in relation to the overall population with diabetes should be highlighted, given the exclusion of advanced late complications in the selection of the patients from the previous study. Finally, it should be stressed that the main objective when the study was designed was not to analyze the association between adherence to the MedDiet and the results reported by the patient with T2DM. However, the use of specific questionnaires designed to measure the main outcome variables and of tools validated to assess intake strengthens the results of the study.

## Conclusion

The results of this study show that in patients with T2DM high adherence to the MedDiet was associated with higher diabetes treatment satisfaction. Moreover, despite the fact that a significant association was not demonstrated with the overall score of HRQoL, a higher adherence to the MedDiet was associated with a better score in some of its dimensions. There is a clear need to undertake new research to analyze the causal relationships existing between adherence to healthy eating patterns, on the one hand, and quality of life and treatment satisfaction in the diabetic patient on the other. It is important to define whether there is a causal relationship or other associated factors in a bidirectional manner between the food and nutrient intake regime and the results reported by the patient with T2DM.

## Abbreviations

ADDQoL: Audit of Diabetes Dependent Quality of Life
DTSQ: Diabetes Treatment Satisfaction Questionnaire
HbA1c: glycated haemoglobin
HDL: high density lipoprotein
HRQoL: Health-Related Quality of Life
LDL: low density lipoprotein
MedDiet: traditional Mediterranean Diet
rMED: relative Mediterranean Diet score
SD: standard deviation
SE: standard error
T2DM: type 2 diabetes mellitus
